# Tetradecylthiopropionic acid induces hepatic mitochondrial dysfunction and steatosis, accompanied by increased plasma homocysteine in mice

**DOI:** 10.1186/s12944-016-0192-9

**Published:** 2016-02-05

**Authors:** Rolf K. Berge, Bodil Bjørndal, Elin Strand, Pavol Bohov, Carine Lindquist, Jan Erik Nordrehaug, Asbjørn Svardal, Jon Skorve, Ottar Nygård

**Affiliations:** Department of Clinical Science, University of Bergen, 5020 Bergen, Norway; Department of Heart Disease, Haukeland University Hospital, 5021 Bergen, Norway; Department of Heart Disease, Stavanger University Hospital, 4142 Stavanger, Norway; KG Jebsen Centre for Diabetes Research, University of Bergen, 5020 Bergen, Norway

**Keywords:** Fatty acid oxidation, Fatty liver disease, Homocysteine, One-carbon metabolism, Carnitine

## Abstract

**Background:**

Hepatic mitochondrial dysfunction plays an important role in the pathogenesis of non-alcoholic fatty liver disease (NAFLD). Methyl donor supplementation has been shown to alleviate NAFLD, connecting the condition to the one-carbon metabolism. Thus, the objective was to investigate regulation of homocysteine (Hcy) and metabolites along the choline oxidation pathway during induction of hepatic steatosis by the fatty acid analogue tetradecylthiopropionic acid (TTP), an inhibitor of mitochondrial fatty acid oxidation.

**Methods:**

Mice were fed a control diet, or diets containing 0.3 %, 0.6 %, or 0.9 % (w/w) TTP for 14 days. Blood and liver samples were collected, enzyme activities and gene expression were analyzed in liver, lipid and fatty acid composition in liver and plasma, one-carbon metabolites, B-vitamin status, carnitine and acylcarnitines were analyzed in plasma.

**Results:**

Liver mitochondrial fatty acid oxidation decreased by 40 % and steatosis was induced in a dose dependent manner; total lipids increased 1.6-fold in animals treated with 0.3 % TTP, 2-fold with 0.6 % TTP and 2.1 fold with 0.9 % TTP compared to control. The higher hepatic concentration of fatty acids was associated with shortening of carbon-length. Furthermore, the inhibited fatty acid oxidation led to a 30-fold decrease in plasma carnitine and 9.3-fold decrease in acetylcarnitine at the highest dose of TTP, whereas an accumulation of palmitoylcarnitine resulted. Compared to the control diet, TTP administration was associated with elevated plasma total Hcy (control: 7.2 ± 0.3 umol/L, 0.9 % TTP: 30.5 ± 5.9 umol/L) and 1.4-1.6 fold increase in the one-carbon metabolites betaine, dimethylglycine, sarcosine and glycine, accompanied by changes in gene expression of the different B-vitamin dependent pathways of Hcy and choline metabolism. A positive correlation between total Hcy and hepatic triacylglycerol resulted.

**Conclusions:**

The TTP-induced inhibition of mitochondrial fatty acid oxidation was not associated with increased hepatic oxidative stress or inflammation. Our data suggest a link between mitochondrial dysfunction and the methylation processes within the one-carbon metabolism in mice.

## Background

The liver is important in regulating lipid homeostasis and metabolism [1] and under normolipidemic conditions all fat to the liver is either oxidized or secreted as very low-density lipoprotein-triacylglycerols (VLDL-TAGs) and finally stored in white adipose tissue. However, the prevalence of excessive accumulation of hepatic fat unrelated to alcohol consumption (hepatic steatosis or non-alcoholic fatty liver disease (NAFLD)) has increased in adults of industrialized countries [2]. Moreover, its development often parallels that of obesity-associated diseases like insulin resistance, metabolic syndrome, dyslipidemia, and type 2 diabetes mellitus, and there is a strong association between NAFLD and risk of CVD [3].

Over time, an excess uptake of lipids to the liver will require an adaptive increase in mitochondrial oxidative capacity. An increased exposure to oxidative stress and inflammation may lead to dysregulated mitochondrial function, which is central for the intrahepatic accumulation of lipids [4, 5]. Reduced fatty acid oxidation, as well as increased adipose tissue lipolysis and liver lipid uptake can cause excessive accumulation of hepatic TAG. Peripheral insulin resistance will lead to hyperglycemia and increased hepatic lipogenesis, while hyperinsulinemia will inhibit lipoprotein synthesis and secretion [6]. The antioxidant capacity of the diet could potentially play a vital role in the development of NAFLD and the prevention of CVD-risk [7], in part by maintaining mitochondrial function.

Tetradecylthiopropionic acid (TTP) is a synthetic saturated fatty acid (SFA) with a sulphur atom in the 4-position from the carboxylic end. This implies that the fatty acid derivative can undergo only one cycle of mitochondrial β-oxidation [8]. The weak peroxisomal proliferator fatty acid TTP will then induce fatty liver by the accumulation of alkylthioacryloyl-CoA, which is believed to inhibit carnitine palmitoyl transferase 2 (CPT II) [8–10]. In addition, an accelerated TAG biosynthesis due to stimulation of the enzyme phosphatidate phosphohydrolase has been proposed [11, 12].

**Fig. 1:**
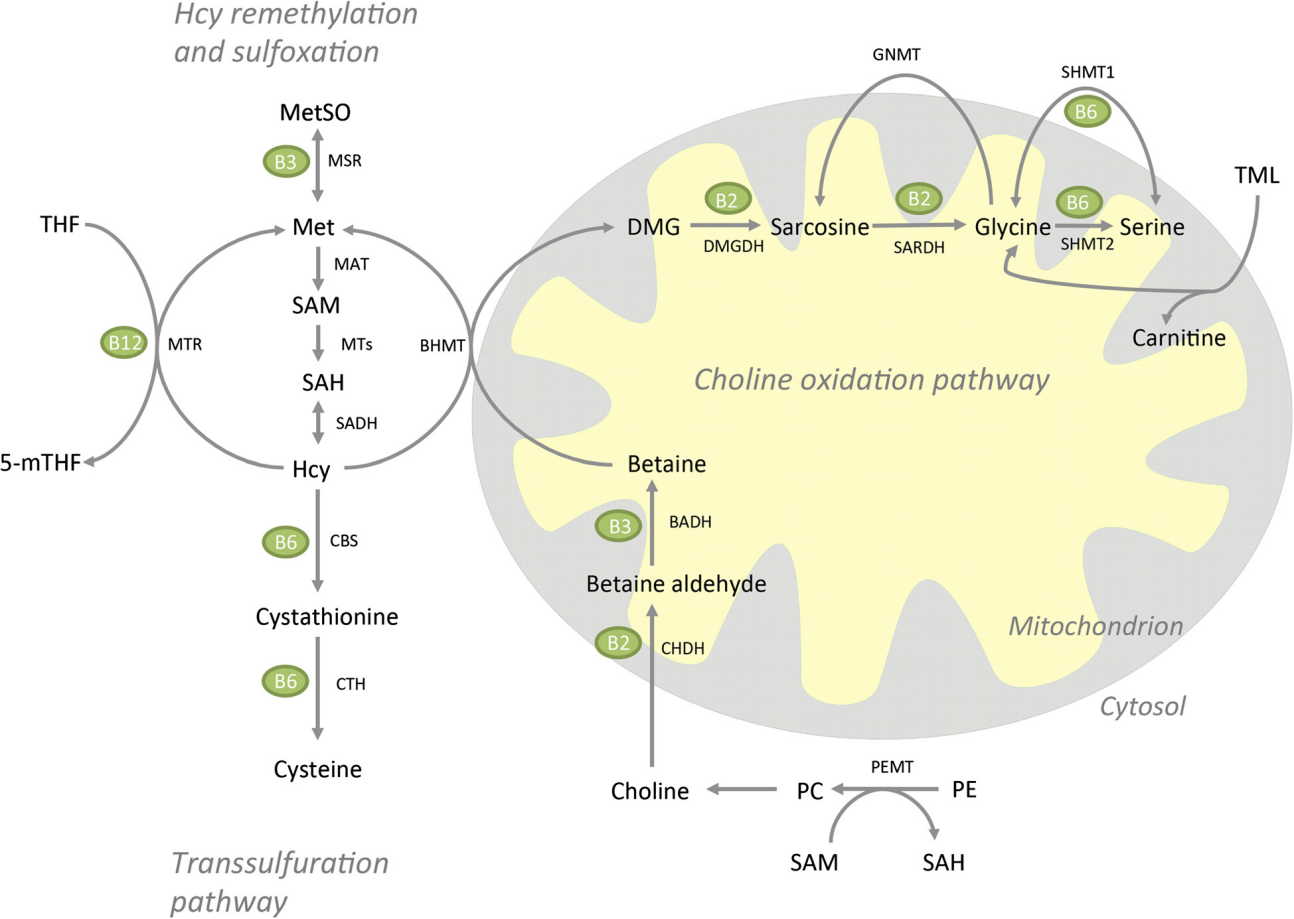
Metabolites of the homocysteine remethylation and sulfoxation, transsulfuration, and choline oxidation pathways. Names of enzymes are beside the arrows. Vitamins involved in the enzymatic reactions are shown in green circles. Abbreviations: BADH, betaine aldehyde dehydrogenase; BHMT, betaine-homocysteine methyltransferase; CBS, cystathionine beta-synthase; CHDH, choline dehydrogenase; CTH, cystathionine gamma-lyase; DMG, dimethylglycine; DMGDH, dimethylglycine dehydrogenase; GNMT, glycine N-methyltransferase; Hcy, homocysteine; MAT, methionine adenosyltransferase; Met, methionine; MetSO, methionine sulfoxide; 5-mTHF, 5-methyltetrahydrofolate; MTs, methyltransferases; MTR, methionine synthase; MSR (encoded by the gene *Mtrr*), methionine synthase reductase; PC, phosphatidylcholine; PE, phosphatidyletanolamine; PEMT, phosphatidyl-ethanolamine methyltransferase; SAH, s-adenosylhomocysteine; SAM, s-adenosylmethionine; SARDH, sarcosine dehydrogenase; SHMT1, serine hydroxymethyltransferase 1; SHMT2, serine hydroxymethyltransferase 2; THF, tetrahydrofolate; TML, trimethyllysine

We have recently shown that elevated plasma choline [13] and dimethylglycine (DMG) [14, 15] are associated with an increased risk of acute myocardial infarction. Plasma total homocysteine (tHcy) is a well-known risk marker for CVD and hyperhomocysteinemia is associated with alterations in intracellular lipid metabolism [16]. Moreover, during oxidative stress the one-carbon cycle shifts from methylation pathways to the transsulfuration pathway [17]. Previous knowledge regarding the influence of TTP-induced fatty liver on the circulating levels of metabolites along the choline oxidation pathway is scarce. The choline oxidation pathway is a vital part of Hcy metabolism and could potentially be affected by mitochondrial dysfunction as it is located in the mitochondrial compartment. Since a dietary deficiency in methyl donors may lead to fatty liver [18], we aimed to investigate how tHcy and metabolites along the choline oxidation pathway were regulated during TTP-induced fatty liver (Fig. 1). The present study suggests that reduced mitochondrial function and fatty liver is associated with low carnitine and acetylcarnitine concentrations and elevated plasma palmitoylcarnitine. Oxidative stress was, however, not increased. In addition, TTP resulted in elevated plasma tHcy through interfering with all three vitamin B-dependent pathways of Hcy metabolism, including transsulfuration.

## Results

### Effect on body and liver weights and feed intake

C57BL/6 mice were fed a control diet containing 7 % (w/w) fat for two weeks, or the same diet supplemented with 0.3, 0.6 or 0.9 % TTP. Mice fed the TTP diets had a significantly lower feed intake than mice fed the control diet, and this was accompanied by a significantly lower body weight (Table 1). The hepatic index (ratio liver weight to body weight) was, however, unchanged. Alanine transaminase (ALT) in plasma tended to increase, but these data were not statistically significant.

**Table 1:** Body weight, organ weight, feed intake, and plasma lipids in control and TTP-treated mice^a^

	Diet groups			
	Control	0.3 % TTP	0.6 % TTP	0.9 % TTP
Body weight^b^ (g)	28.7 ± 1.1	23.8 ± 1.3***	22.5 ± 0.8***	22.4 ± 1.3***
Feed intake^c^ (g)	4.2 ± 0.3	3.3 ± 0.7*	3.1 ± 0.9**	3.2 ± 0.4*
Hepatic index^d^ (%)	4.6 ± 0.7	4.5 ± 0.6	5.1 ± 0.7	5.0 ± 0.8
Plasma ALT (u/L)	29.7 ± 2.6	38.9 ± 25.8	31.8 ± 8.9	32.7 ± 6.9
Plasma TAG (mmol/L)	0.63 ± 0.11	0.60 ± 0.15	0.65 ± 0.17	0.51 ± 0.15
Plasma chol (mmol/L)	2.67 ± 0.41	2.62 ± 0.41	2.74 ± 0.23	3.08 ± 0.28
Plasma PL (mmol/L)	2.75 ± 0.35	2.62 ± 0.34	2.68 ± 0.25	2.75 ± 0.27
Plasma NEFA (mmol/L)	0.16 ± 0.06	0.37 ± 0.13	0.39 ± 0.30	0.50 ± 0.20**
Plasma glucose (mmol/L)	14.8 ± 2.1	12.1 ± 1.3*	11.0 ± 2.1***	11.2 ± 1.0**

*Abbreviations*: *ALT* alanine aminotransferase; *NEFA* non-esterified fatty acids; *TTP* tetradecylthiopropionic acid; *TAG* triacylglycerol; *PL* phospholipids; *Chol*, cholesterol

^a^Values are means with standard deviation (*n* = 7–8), and values statistically different from control were determined by one-way ANOVA with Dunnett’s post hoc test (*P < 0.05, **P < 0.01, ***P < 0.001)

^b^Body weight after two weeks of treatment

^c^Average feed intake per mouse per day calculated from measurements every 3 days throughout the experiment (*n* = 3)

^d^Hepatic index was calculated as percentage of liver weight to body weight

### Hepatic fatty liver

**Fig. 2:**
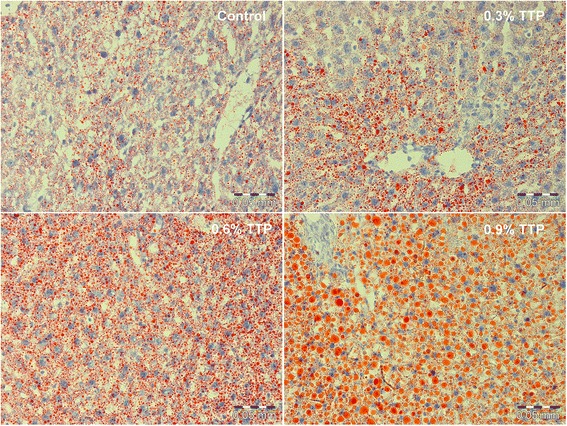
Lipid droplet morphology in frozen liver tissue sections from tetradecylthiopropionic acid (TTP) treated mice. Representative images of Oil-red-O stained liver sections from male C57BL/6 mice fed a control low-fat diet, or low-fat diets supplemented with 0.3 % (w/w), 0.6 % or 0.9 % TTP for 2 weeks (*n* = 3)

**Fig. 3:**
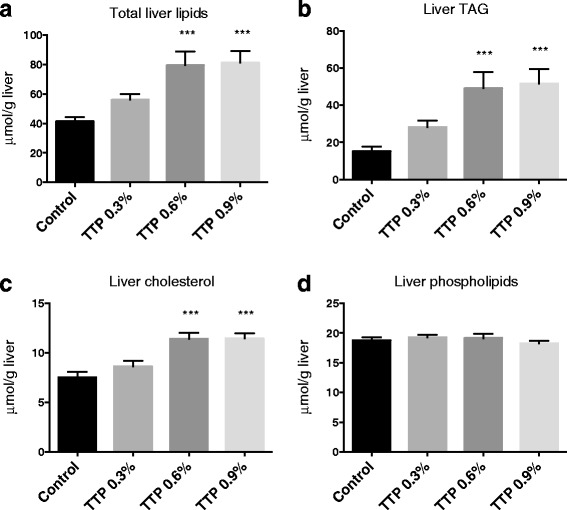
Hepatic lipid profile with increasing dose of tetradecylthiopropionic acid (TTP). Male C57BL/6 mice were fed a low-fat control diet (black), or low-fat diets supplemented with 0.3 % (grey) (w/w), 0.6 % (dark grey) or 0.9 % (light grey) TTP for 2 weeks. Levels of (**a**) total hepatic lipids (combination of triacylglycerol (TAG), cholesterol, and phospholipids), (**b**) TAG, (**c**) cholesterol, and (**d**) phospholipids were as indicated. Mean values with SD are presented (*n* = 7-8), and statistically significant difference from control was determined by one-way ANOVA with Dunnett’s post hoc test (*P < 0.05, **P < 0.01, ***P < 0.001)

There was increased lipid droplet accumulation in hepatocytes after TTP treatment, resulting in abnormalities in liver morphology (Fig. 2). Moreover, the total liver lipids increased 2-fold and 2.1-fold at dosages 0.6 and 0.9 % of TTP, respectively (Fig. 3a). This was due to an increase in TAG (Fig. 3b) and cholesterol (Fig. 3c), while hepatic phospholipid levels were not changed in TTP-treated mice compared to controls (Fig. 3d). The higher hepatic concentration of fatty acids (data not shown) was mainly due to increased wt % of MUFAs, while SFAs and n-6 PUFAs were significantly decreased and n-3 PUFAs were unchanged. The decreased relative amount of n-6 PUFAs was due to decreased wt % of long chain fatty acids, reflecting a decreased content of C20:4n-6 (arachidonic acid, AA), C20:2n-6, C20:3n-6 and C22:5n-6, while C18:2n-6 (linoleic acid, LA) was unchanged (data not shown). It is worth noting that C18:3n-3 (α-linolenic acid, ALA) and C18:4n-3 was significantly increased at the lowest TTP dose, whereas C20:5n-3 (eicosapentaenoic acid, EPA), C22:5n-3 (docosapentaenoic acid, DPA), and C22:6n-3 (docosahexaenoic acid, DHA) was unchanged after TTP treatment. As a result, the ratio of n-3 to n-6 PUFAs was higher in TTP-treated animals. The n-9 PUFAs were unchanged by TTP administration (data not shown), but most of the long-chain MUFAs (C20-C24), both of the n-7 and n-9 family, decreased. Thus, the increase in total MUFAs was due to the relative higher amount of C18:1n-9, C16:1n-7, and C16:1n-9 in TTP-treated mice (Table 2). Moreover, it was of interest that the relative amount of long-chain SFAs such as C18:0, C20:0, C22:0 and C24:0 was decreased, whereas the content of medium chain SFAs as C12:0 and C14:0 was increased (Table 2). C16:0 was not changed by TTP treatment (data not shown).

**Table 2:** Comparison of hepatic fatty acid composition in control and TTP-treated mice

	Diet groups			
Fatty acid^a^ (wt %)	Control	0.3 % TTP	0.6 % TTP	0.9 % TTP
Σ SFA	32.0 ± 2.8	31.1 ± 1.5	29.3 ± 1.8*	29.0 ± 1.7**
C12:0	0.01 ± 0.003	0.05 ± 0.02*	0.05 ± 0.02**	0.05 ± 0.02**
C14:0	0.31 ± 0.09	0.53 ± 0.14**	0.69 ± 0.19***	0.71 ± 0.17***
C18:0	10.3 ± 2.4	8.5 ± 1.4	6.6 ± 2.1**	6.7 ± 1.0**
C20:0	0.23 ± 0.05	0.14 ± 0.05***	0.07 ± 0.03***	0.06 ± 0.01***
C22:0	0.29 ± 0.07	0.21 ± 0.06*	0.11 ± 0.07***	0.10 ± 0.04***
C24:0	0.22 ± 0.06	0.13 ± 0.02**	0.10 ± 0.05***	0.09 ± 0.04***
Σ MUFAs	27.0 ± 5.1	29.7 ± 3.7	39.6 ± 8.8**	41.5 ± 5.1***
C16:1n-7	3.0 ± 0.9	3.6 ± 0.3	4.7 ± 1.5*	4.9 ± 1.3*
C16:1n-9	0.38 ± 0.14	0.58 ± 0.06*	0.82 ± 0.21***	0.93 ± 0.18***
C18:1n-9	19.5 ± 5.1	22.6 ± 2.2	29.9 ± 6.4***	31.8 ± 4.1***
C20:1n-7	0.18 ± 0.07	0.09 ± 0.02**	0.09 ± 0.05**	0.06 ± 0.03***
C22:1n-7	0.05 ± 0.01	0.02 ± 0.01***	0.01 ± 0.01***	0.01 ± 0.01***
C24:1n-9	0.27 ± 0.06	0.19 ± 0.03*	0.16 ± 0.06***	0.14 ± 0.04***
Σ n-6 PUFAs	30.6 ± 3.7	27.8 ± 1.5	21.9 ± 6.1***	20.7 ± 3.0***
C20:2n-6	0.25 ± 0.07	0.20 ± 0.03	0.18 ± 0.05*	0.17 ± 0.03*
C20:3n-6	1.7 ± 0.2	1.2 ± 0.1***	0.9 ± 0.4***	1.0 ± 0.2***
C20:4n-6	13.4 ± 2.1	10.5 ± 1.5**	6.8 ± 2.6***	6.8 ± 1.2***
C22:5n-6	0.42 ± 0.29	0.18 ± 0.04*	0.12 ± 0.04**	0.13 ± 0.03**
Σ n-3 PUFAs	10.0 ± 0.9	11.1 ± 1.4	8.9 ± 3.1	8.5 ± 1.6
C18:3n-3	0.26 ± 0.06	0.51 ± 0.12*	0.48 ± 0.24*	0.51 ± 0.20*
C18:4n-3	0.02 ± 0.01	0.06 ± 0.02	0.07 ± 0.05*	0.07 ± 0.05*
C20:5n-3	0.39 ± 0.09	0.46 ± 0.07	0.43 ± 0.20	0.40 ± 0.15
C22:5n-3	0.28 ± 0.06	0.44 ± 0.04*	0.40 ± 0.20	0.39 ± 0.16
C22:6n-3	8.9 ± 0.8	9.5 ± 1.5	7.4 ± 2.7	7.0 ± 1.2
Σ n-3 PUFA/Σ n-6 PUFA^b^	0.33 ± 0.02	0.40 ± 0.04**	0.40 ± 0.06**	0.41 ± 0.04**

*Abbreviations*: *SFAs* saturated fatty acids; *MUFAs* monounsaturated fatty acids; *PUFAs* polyunsaturated fatty acids; *TTP* tetradecylthiopropionic acid

^a^Means with standard deviation are shown (*n* = 7–8), and results were analyzed by one-way ANOVA with Dunnett’s post hoc test (P* < 0.05, P** < 0.01, P*** < 0.001)

^b^n-3 PUFA/n-6 PUFA calculated as: (18:4n-3 + 18:3n-3 + 20:3n-3 + 20:4n-3 + 21:5n-3 + 22:6n-3 + 22:5n-3)/(18:3n-6 + 18:2n-6 + 20:4n-6 + 20:3n-6 + 20:2n-6 + 22:5n-6 + 22:4n-6 + 22:2n-6)

### Hepatic fatty acid oxidation and plasma carnitine derivatives

**Fig. 4:**
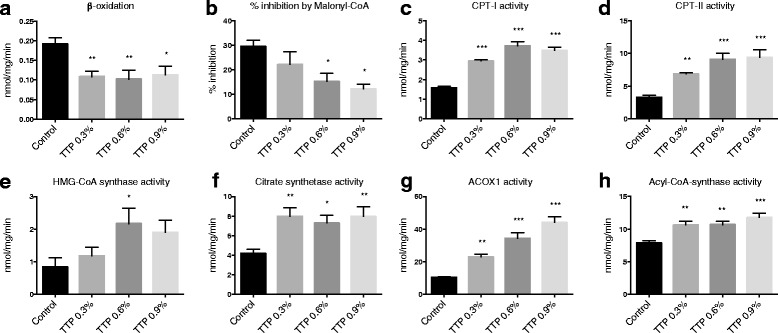
The effect of tetradecylthiopropionic acid (TTP) on hepatic lipid metabolism in mice. Male C57BL/6 mice were fed a low-fat control diet (black), or low-fat diets supplemented with 0.3 % (grey) (w/w), 0.6 % (dark grey) or 0.9 % (light grey) TTP for 2 weeks. **a** β-oxidation as measured by degradation of palmitoyl-CoA in fresh liver homogenates. **b** Inhibition of the β-oxidation reaction in the presence of malonyl-CoA. The activities of (**c**) Carnitine palmitoyl transferase (CPT)-I, (**d**) CPT-II, (**e**) HMG-CoA synthase, (**f**) citrate synthetase, (**g**) fatty acyl-CoA oxidase (ACOX1), and (**h**) acyl-CoA synthase were measured in frozen liver homogenates. Mean values with SD are presented (**a**–**c**: *n* = 5 – 7, **d**–**i**: *n* = 7–8), and statistically significant difference from control was determined by one-way ANOVA with Dunnett’s post hoc test (*P < 0.05, **P < 0.01, ***P < 0.001)

The hepatic mitochondrial β-oxidation, measured as acid soluble products from palmitoyl-CoA (Fig. 4a) was decreased in fresh liver homogenates from TTP-treated mice. The sensitivity to malonyl-CoA-inhibition was reduced in TTP-treated mice compared to controls (Fig. 4b). Unexpectedly, the activities of mitochondrial CPT I and II were increased by TTP treatment (Fig. 4c, d), as was the HMG-CoA synthase activity (Fig. 4e). Thus, enzymes involved in both import of fatty acids to the mitochondria and ketone body production was increased by TTP. Moreover, the hepatic citrate synthase activity, often used as a matrix enzyme marker for intact mitochondria, was increased in mice treated with TTP (Fig. 4f). Furthermore, hepatic fatty acyl-CoA oxidase 1 (ACOX1) activity increased in a dose-dependent manner suggesting an induction of the peroxisomal β-oxidation system (Fig. 4g). The acyl-CoA synthetase (ACSL) activity was also increased by TTP administration (Fig. 4h). The activities of fatty acid synthase (FAS), an enzyme involved in lipogenesis, and glycerol-3-phosphate acyltransferase (GPAT), an enzyme involved in TAG biosynthesis, were unchanged (data not shown).

Gene expression of fatty acid binding protein (*Fabp1*), peroxisome proliferator-activated receptor gamma (*Pparg*), PPAR gamma coactivator 1 alpha (*Ppargc1a*), *CD36* and hepatic hormone sensitive lipase (*Lipe*) were all increased after TTP treatment (Table 3). The mRNA levels of hepatic lipase (*Lipc*), PPAR alpha (*Ppara*), ATP citrate lyase, acylcarnitine translocase and *ApoB* remained constant (Table 3).

**Table 3:** Hepatic gene expression levels^a^

Gene symbol	Gene name	Control diet	0.9 % TTP diet	*P*-value
PPAR co-activators:				
*Pparg1a*	Peroxisome proliferator-activated receptor gamma, coactivator 1 alpha	1.00 ± 0.32	1.56 ± 0.33	0.021
*Ppara*	Peroxisome proliferator-activated receptor alpha	1.00 ± 0.37	1.12 ± 0.23	0.468
*Pparg*	Peroxisome proliferator-activated receptor gamma, coactivator 1 beta	1.00 ± 0.78	1.57 ± 0.31	0.040
Fatty acid mobilization, uptake, and secretion:				
*CD36*	Peroxisome proliferator-activated receptor gamma, coactivator 1 beta	1.00 ± 0.64	3.24 ± 0.67	<0.001
*Lipe*	Hormone sensitive lipase	1.00 ± 0.16	1.24 ± 0.24	0.036
*Lipc*	Hepatic lipase	1.00 ± 0.14	0.93 ± 0.15	0.384
*Fabp1*	Fatty acid binding protein 1, liver	1.00 ± 0.35	1.38 ± 0.27	0.039
*Acly*	ATP citrate lyase	1.00 ± 0.22	0.97 ± 1.02	0.187
*Apob*	Apolipoprotein B	1.00 ± 0.16	0.94 ± 0.14	0.450
*Slc25a20*	acylcarnitine translocase	1.00 ± 0.25	1.31 ± 0.44	0.116
Antioxidant activity and inflammation:				
*Tnfa*	Tumor necrosis factor alpha	1.00 ± 0.44	0.51 ± 0.09	0.014
*Il1b*	Interleukin 1, beta	1.00 ± 0.63	0.68 ± 0.32	0.054
*Sod1*	Superoxide dismutase 1, soluble	1.00 ± 0.11	1.27 ± 0.22	0.010
*Sod2*	Superoxide dismutase 2, mitochondrial	1.00 ± 0.12	1.08 ± 0.11	0.187
Phosphatidyl synthesis and metabolism:				
*Chka*	Choline kinase alpha	1.00 ± 0.35	0.35 ± 0.14	<0.001
*Chkb*	Ethanolamine kinase/Choline kinase beta	1.00 ± 0.18	1.49 ± 0.29	0.001
*Pcyt1a*	Phosphate cytidylyltransferase 1, choline, alpha	1.00 ± 0.19	0.80 ± 0.08	0.018
Choline oxidation pathway, mitochondria:				
*Chdh*	Choline dehydrogenase	1.00 ± 0.35	1.01 ± 0.15	0.941
*Dmgdh*	Dimethylglycine dehydrogenase precursor	1.00 ± 0.28	0.65 ± 0.07	0.007
*Sardh*	Sarcosine dehydrogenase	1.00 ± 0.31	0.69 ± 0.14	0.032
*Shmt2*	Serine hydroxymethyltransferase 2 (mit)	1.00 ± 0.24	0.59 ± 0.10	0.001
Choline oxidation pathway, cytosol:				
*Bhmt*	betaine-homocysteine S-methyltransferase	1.00 ± 0.76	0.15 ± 0.05	0.012
*Gnmt*	Glycine N-methyltransferase	1.00 ± 0.14	0.90 ± 0.15	0.232
Amino acid metabolism and derivatives:				
*Mtr/Mr*	Methionine synthase (5-methyltetrahydrofolate-homocystein methyltransferase)	1.00 ± 0.22	0.70 ± 0.12	0.006
*Mtrr/Msr*	Methionine synthase reductase (5-methyltetrahydrofolate-homocystein)	1.00 ± 0.50	0.68 ± 0.17	0.040
*Msra*	Methionine sulfoxide reductase A	1.00 ± 0.50	0.88 ± 0.21	0.556
*Msrb2*	Methionine sulfoxide reductase B2	1.00 ± 0.10	0.75 ± 0.14	0.001
*Shmt1*	Serine hydroxyl-methyltransferse (soluble)	1.00 ± 0.57	0.66 ± 0.23	0.165
Transsulfuration:				
*Cbs*	Cystathionine-beta-synthase	1.00 ± 0.46	0.49 ± 0.18	0.017
*Cth*	Cystathionase (cystathionine gamma lyase)	1.00 ± 0.51	0.63 ± 0.22	0.121

*Abbreviations*: *TTP* tetradecylthiopropionic acid

^a^Expression levels were normalized to 18 s expression and relative values to control are given as means ± standard deviation (*n* = 7–8). Results were analyzed by unpaired *t*-test, and *P* –values < 0.05 were considered significant

**Fig. 5:**
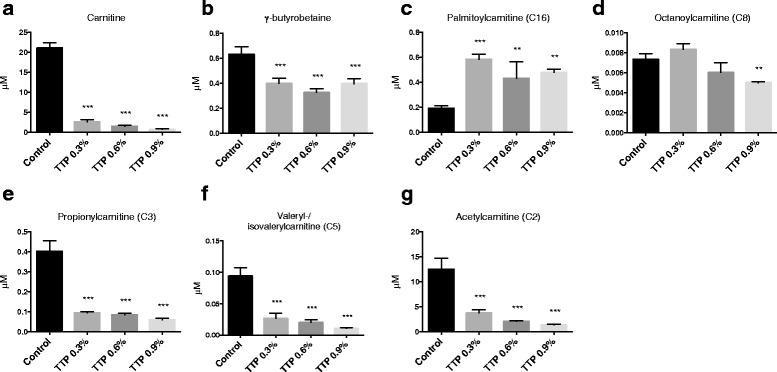
The effect of tetradecylthiopropionic acid (TTP) on plasma L-carnitine precursors, free L-carnitine, and L-carnitine esters. Male C57BL/6 mice were fed a low-fat control diet (black), or low-fat diets supplemented with 0.3 % (grey) (w/w), 0.6 % (dark grey) or 0.9 % (light grey) TTP for 2 weeks, and (**a**) L-carnitine, (**b**) γ-butyrobetaine, (**c**) palmitoylcarnitine, (**d**) octanoylcarnitine, (**e**) propionylcarnitine, (**f**) (iso)valerylcarnitine, and (**g**) acetylcarnitine was measured in fasting plasma samples. Mean values with SD are presented (pooled samples from 2-3 mice, *n* = 3), and statistically significant difference from control was determined by one-way ANOVA with Dunnett’s post hoc test (*P < 0.05, **P < 0.01, ***P < 0.001)

L-carnitine is essential for mitochondrial oxidation of long-chain fatty acids, and the plasma concentrations of L-carnitine and its precursor γ-butyrobetaine were reduced in mice treated with TTP (Fig. 5a, b). The highest dose of TTP gave the most prominent effect on L-carnitine, reducing its level 30-fold compared to control. Among plasma acylcarnitines, the long-chain palmitoylcarnitine was increased (Fig. 5c), and the medium-chain octanoylcarnitine was reduced by 0.9 % TTP (Fig. 5d). The short-chain acylcarnitines propionyl-, (iso)valeryl-, and acetylcarnitine were decreased in all TTP groups (Fig. 5e-g), and a 9.3-fold reduction in acetylcarnitine was observed in the 0.9 % TTP group compared to control.

### Plasma lipids and fatty acid composition

The plasma concentrations of TAG, cholesterol and phospholipids were not changed by TTP treatment (Table 1). Interestingly, the plasma level of NEFA increased in a dose-dependent manner with TTP. There were no changes in plasma wt % of SFAs, MUFAs and n-6 PUFAs in TTP-treated mice, and only a small increase in n-3 PUFAs in the 3 % TTP group (Table 4). Between-group differences in plasma fatty acids were less striking compared to hepatic fatty acids (Table 2), with the exception of a lower level of plasma 22:5n-6 in TTP animals. Altogether, this resulted in an increased ratio of n-3 to n-6 PUFAs (Table 4).

**Table 4:** Plasma fatty acid composition in control and TTP-treated mice^a^

	Diet groups			
Fatty acid (wt %)	Control	0.3 % TTP	0.6 % TTP	0.9 % TTP
Σ SFAs	32.1 ± 1.5	31.8 ± 0.9	31.2 ± 0.6	30.9 ± 0.6
Σ MUFAs	22.7 ± 2.2	20.6 ± 3.1	23.8 ± 4.8	22.2 ± 2.4
Σ n-6 PUFAs	36.9 ± 1.1	37.8 ± 2.1	35.0 ± 3.9	36.7 ± 2.1
C22:5n-6	0.29 ± 0.05	0.16 ± 0.04***	0.14 ± 0.06***	0.14 ± 0.04***
Σ n*-*3 PUFAs	7.8 ± 0.5	9.2 ± 0.8*	8.9 ± 1.7	8.7 ± 0.4
C20:5n-3	0.53 ± 0.07	0.62 ± 0.06	0.69 ± 0.22	0.70 ± 0.16
C22:5n-3	0.24 ± 0.03	0.31 ± 0.04	0.32 ± 0.11	0.33 ± 0.09
C22:6n-3	6.6 ± 0.5	7.7 ± 0.9	7.3 ± 1.4	7.2 ± 0.4
Σ n-3 PUFA/Σ n-6 PUFA ratio^b^	0.21 ± 0.02	0.24 ± 0.02*	0.25 ± 0.03**	0.24 ± 0.01*

*Abbreviations*: *SFAs* saturated fatty acids; *MUFAs* monounsaturated fatty acids; *PUFAs* polyunsaturated fatty acids; *TTP* tetradecylthiopropionic acid

^a^Means with standard deviation are shown (*n* = 7–8), and results were analyzed by one-way ANOVA with Dunnett’s post hoc test (P* < 0.05, P** < 0.01, P*** < 0.001)n-3

^b^PUFA/n-6 PUFA calculated as: (18:4n-3 + 18:3n-3 + 20:3n-3 + 20:4n-3 + 21:5n-3 + 22:6n-3 + 22:5n-3)/(18:3n-6 + 18:2n-6 + 20:4n-6 + 20:3n-6 + 20:2n-6 + 22:5n-6 + 22:4n-6 + 22:2n-6)

### Antioxidant status and inflammation

**Fig. 6:**
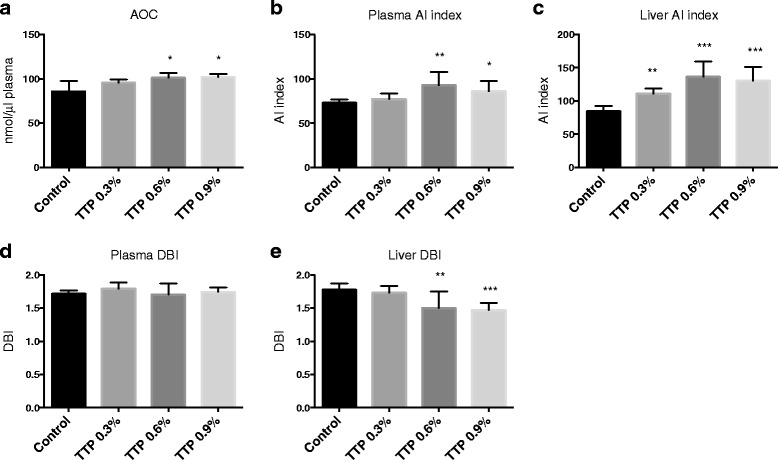
The effect of tetradecylthiopropionic acid (TTP) on antioxidative and anti-inflammatory parameters. Male C57BL/6 mice were fed a low-fat control diet (black), or low-fat diets supplemented with 0.3 % (grey) (w/w), 0.6 % (dark grey) or 0.9 % (light grey) TTP for 2 weeks. Levels of (**a**) plasma total antioxidant capacity, (**b**) plasma anti-inflammatory fatty acid index, (**c**) liver anti-inflammatory fatty acid index, (**d**) plasma double bond index (DBI), (**e**) liver DBI were as indicated. Mean values with SD are presented (**a**: *n* = 3–8; **b**–**e**: *n* = 7–8), and statistically significant difference from control was determined by one-way ANOVA with Dunnett’s post hoc test (*P < 0.05)

During oxidative stress, fatty acids shorten in chain length and decrease in unsaturation [17]. The plasma total antioxidant capacity (Fig. 6a) significantly increased after TTP administration. The fatty acid inflammatory index increased in plasma (Fig. 6b) and liver (Fig. 6c), and this was accompanied by significantly decreased hepatic gene expression of IL-1β and TNFα after TTP treatment (Table 3). The double bond index (DBI) was unchanged in plasma (Fig. 6d), but reduced in liver (Fig. 6e), and the hepatic mRNA levels of cytosolic superoxide dismutase 1 (*Sod1*) and mitochondrial *Sod2* were increased and remained constant, respectively (Table 3).

### Plasma metabolites in methylation pathways

**Fig. 7:**
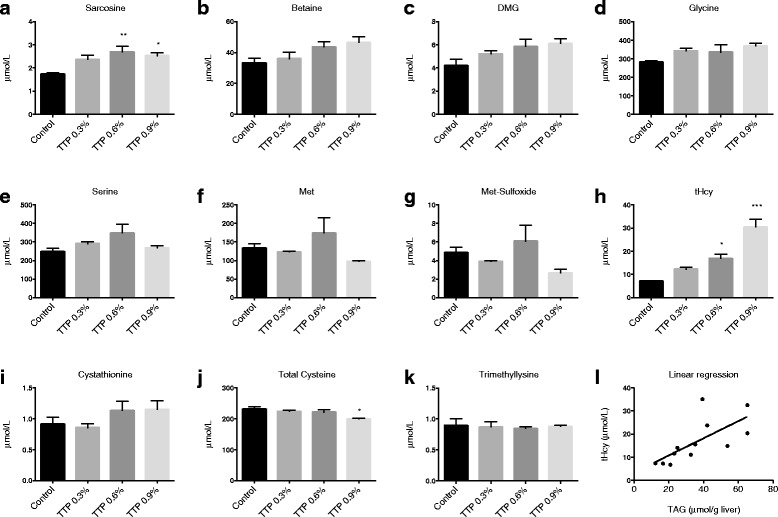
The effect of tetradecylthiopropionic acid (TTP) on plasma one-carbon metabolites in mice. Male C57BL/6 mice were fed a low-fat control diet (black), or low-fat diets supplemented with 0.3 % (grey) (w/w), 0.6 % (dark grey) or 0.9 % (light grey) TTP for 2 weeks, and (**a**) sarcosine, (**b**) betaine, (**c**) dimethylglycine (DMG), (**d**) glycine, (**e**) serine, (**f**) methionine (Met), (**g**) Met-sulfoxide, (**h**) total homocysteine (tHcy), (**i**) cystathionine, (**j**) total cysteine, and (**k**) trimethyllysine was measured in fasting plasma samples. Mean values with SD are presented (pooled samples from 2–3 mice, *n* = 3), and statistically significant difference from control was determined by one-way ANOVA with Dunnett’s post hoc test (*P < 0.05, **P < 0.01). **l** There was a positive association between hepatic TAG and plasma tHcy as demonstrated by linear regression

No changes in the plasma concentrations of choline were observed in mice fed TTP compared to controls (data not shown). The plasma concentration of sarcosine (Fig. 7a) was increased 1.6-fold in the 0.6 % TTP-treated mice compared to control-fed mice, while betaine, DMG and glycine tended to increase in a dose-dependent manner [*t*-test control vs 0.9 % TTP was *P* = 0.047 (Fig. 7b), *P* = 0.049 (Fig. 7c), *P* = 0.007 (Fig. 7d)]. The serine level was unchanged (Fig. 7e).

The plasma levels of methionine (Fig. 7f) and methionine sulfoxide (Fig. 7g) were unchanged by TTP, whereas tHcy (Fig. 7h) increased in a dose-dependent manner in TTP-fed mice (control: 7.2 ± 0.3 umol/L, 0.9 % TTP: 30.5 ± 5.9 umol/L). The transsulfuration pathway was also affected by TTP treatment, as the plasma content of cysteine was decreased compared to control (Fig. 7j). The plasma cystathionine level was unchanged (Fig. 7i). The plasma level of the L-carnitine precursor trimethyllysine was not significantly changed after TTP treatment (Fig. 7k). Interestingly, there was a strong correlation between plasma tHcy and hepatic TAG concentration (correlation coefficient, *r* = 0.798, *P* = 0.003; Fig. 7l).

### Plasma vitamin B status

**Fig. 8:**
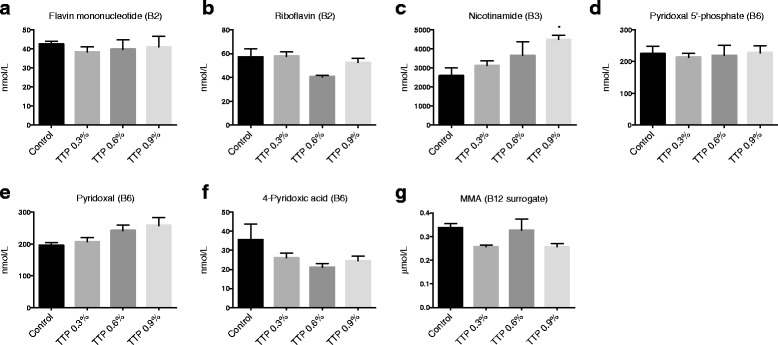
The effect of tetradecylthiopropionic acid (TTP) on plasma B-vitamins and derivatives. Male C57BL/6 mice were fed a low-fat control diet (black), or low-fat diets supplemented with 0.3 % (grey) (w/w), 0.6 % (dark grey) or 0.9 % (light grey) TTP for 2 weeks, and (**a**) flavin mononucleotide, (**b**) riboflavin, (**c**) nicotinamide, (**d**) pyridoxal 5′-phosphate, (**e**) pyridoxal, (**f**) 4-pyridoxic acid, and (**g**) methylmalonic acid (MMA), was measured in fasting plasma samples. Mean values with SD are presented (pooled samples from 2-3 mice, *n* = 3), and statistically significant difference from control was determined by one-way ANOVA with Dunnett’s post hoc test (*P < 0.05)

Several enzymes involved in the choline oxidation, transsulfuration and folate cycle depend on B vitamins as cofactors (Fig. 1). The plasma concentrations of the B2 vitamers flavine mononucleotide and riboflavin were not changed in mice fed the TTP diet (Fig. 8a, b). The plasma concentration of the B3 vitamer nicotinamide was increased (Fig. 8c), while N1-methylnicotinamide was unchanged by the TTP-diet (data not shown). The plasma concentration of pyridoxal 5′ phosphate, the active form of vitamin B6, was unchanged in TTP-fed mice compared to control (Fig. 8d). Plasma pyridoxal tended to increase in a dose-dependent manner (*t*-test control vs 0.9 % TTP: *P* = 0.083; Fig. 8e), and plasma 4-pyridoxic acid was unchanged (Fig. 8f). The biological function of cobalamin is to serve as a cofactor for MS and methylmalonyl-coenzyme A mutase where the final product MMA is utilized as a clinical marker of cobalamin deficiency. It was of interest that the plasma MMA level tended to be lower during TTP-induced fatty liver (Fig. 8g; significant with *t*-test control vs 0.9 % TTP: *P* = 0.025).

### Hepatic gene expression

The regeneration of choline to phosphatidylcholine through the Kennedy pathway is catalysed by choline kinase. In TTP-treated mice the hepatic gene expression of choline kinase alpha (*Chka*) was decreased, while choline kinase beta (*Chkb*) was increased (Table 3). Furthermore, phosphocholine cytidyltransferase alpha (*Pcyt1a*) was significantly decreased. Along the choline oxidation pathway the mRNA level of choline dehydrogenase (*Chdh*) was unchanged. The gene expression of serine hydroxymethyltransferase 2 (*Shmt2*), dimethylglycine dehydrogenase (*Dmgdh*), sarcosine dehydrogenase (*Sardh*), as well as betaine-homocysteine methyltransferase (*Bhmt*), however, was significantly decreased. The mRNA levels of glycine N-methyltransferase (*Gnmt*), transforming glycine to sarcosine, and serine hydroxymethyltransferase 1 (*Shmt1*), transforming cytosolic serine to glycine, were unchanged by TTP administration.

The hepatic gene expression of methionine synthase (*Mtr*) and methionine synthase reductase (*Mtrr*), involved in the folate cycle, was reduced by TTP. In the transsulfuration pathway, there was a significant reduction in the gene expression of cystathionine beta-synthase (*Cbs*) whereas the mRNA level of cystathionine gamma-lyase (*Cth*) was unchanged. Finally, we also observed a significant reduced expression of mitochondrial methionine sulfoxide reductase B2 (*Msrb2*), whereas the expression of methionine sulfoxide reductase A (*Msra*) was unchanged.

## Discussion

The present study demonstrates that inhibition of mitochondrial β-oxidation by TTP administration results in hepatic steatosis, accompanied by changes in plasma L-carnitine derivatives and hepatic fatty acid composition. In addition, changes in the expression of genes regulating plasma levels of metabolites along the B-vitamin dependent pathways of Hcy and choline metabolism, and a 4.3-fold increase plasma tHcy level was observed. Fatty liver and mitochondrial dysfunction was observed despite increased antioxidant activity and anti-inflammatory effects after 2 weeks of TTP treatment.

### TTP and fatty liver

Hepatic accumulation of TAG, cholesterol, and total fatty acids was observed in TTP-treated mice (Fig. 3, Table 2). This was associated with increased plasma concentration of NEFA and an increased gene expression of hepatic *Lipe* and *CD36* (Table 1, 3). Altogether, an increased supply of fatty acids to the liver was probably associated with TTP-induced fatty liver. The flux or draining of fatty acids to the liver was also supported by an increased enzyme activity of fatty acyl-CoA synthetase (Fig. 4h), in parallel with upregulated gene expression of fatty acid binding protein (Table 3). In accordance with previous findings in rats, TTP was associated with inhibited mitochondrial fatty acid oxidation and decreased sensitivity to malonyl-CoA inhibition of CPT I (Fig. 4b) [12, 19]. Previously, a dose of 150 and 300 mg TTP/day/kg body weight resulted in hepatic lipid accumulation in rats [11]. A goal of the current study was to determine the optimal dose of TTP with regard to hepatic steatosis in mice, as mice have a higher metabolic rate than rats. The effect of 0.3, 0.6 or 0.9 % (w/w) TTP in the diet was investigated. Two weeks treatment with both 0.6 and 0.9 % TTP-diets, calculated to a dose of 955 and 1500 mg TTP/day/kg body weight, respectively, caused hepatic lipid accumulation, while the 0.3 % TTP-diet (430 mg TTP/day/kg body weight) did not (Fig. 3). The increase in liver lipids was observed despite a lower feed intake and weight gain in TTP-treated mice compared to control mice. It is possible that the appetite was affected by the TTP-diets; thus, the lack of a pair-fed control group is a limitation of the study. Further investigations are needed to determine whether some findings could be linked to the approximately 20 % lower feed intake in the TTP-groups compared to control. For instance, a reduction in blood glucose was observed in TTP animals. As hypoglycemic effects of drugs inhibiting β-oxidation have previously been observed in rodents [20], the lower glucose levels could also have been caused by a preference for carbohydrate oxidation when β-oxidation is blocked.

In the present study we have shown that the plasma concentrations of both free L-carnitine and γ-butyrobetaine, a precursor of L-carnitine, were decreased in TTP-treated mice (Fig. 5). This was associated with an increased plasma level of the long-chain acylcarnitine palmitoylcarnitine, but no change in the gene expression of acylcarnitine translocase (Fig. 5, Table 3). Thus, the TTP-induced fatty liver resulted in increased consumption and/or reduced biosynthesis of L-carnitine. In addition, the decreased mitochondrial fatty acid oxidation was associated with decreased levels of short-chain acylcarnitines, especially acetylcarnitine. An accumulation of the long-chain acylcarnitine palmitoylcarnitine is in line with an inhibited CPT II activity [8–10]. Medium-chained fatty acids do not depend on the carnitine shuttle for import to the mitochondria. Accordingly, the concentration of octanoylcarnitine was marginally affected in TTP-induced fatty liver.

In parallel with this, the hepatic gene expression of *Pparg* and *Ppargc1a* were upregulated by TTP. PPARγ may be implicated in liver steatosis development, as *Pparg* expression was upregulated in high-fat diet induced liver steatosis in mice, linked to a reduced expression of the PPARγ inhibitor cAMP response element-binding protein (*Creb*) [21, 22]. However, in some studies, PPARγ agonists were able to ameliorate NAFLD, probably through increased β-oxidation [23]. In the current study, increased activities of CPT I and II and ACOX1 were observed. The data suggest that the increased activities of mitochondrial and peroxisomal enzymes probably serve as a compensatory mechanism to TTP-inhibition of mitochondrial β-oxidation. Indeed, in the case of L-carnitine deficiency, a compensatory increase in CPT I was observed [24]. Dysregulated lipogenesis and TAG biosynthesis were probably not involved in TTP-induced steatosis-development as there was no difference between groups in gene expression of ATP citrate lyase (Table 3) or the activities of FAS and GPAT. Production of VLDL did not seem to be influenced, as *ApoB* gene expression remained constant. Thus, hepatic steatosis was probably induced when the amount of imported fatty acids exceeded the catabolism in hepatocytes.

TTP treatment induced changes in liver and plasma fatty acid composition, including an increased n-3 to n-6 PUFA ratio in both liver and plasma. We have previously reported that EPA, but not DHA, is efficiently metabolized through mitochondrial oxidation of fatty acids. DHA is, however, a good substrate for the peroxisomal fatty acid oxidation system, which is also active in chain shortening of fatty acids [25]. Accordingly, in a situation with impaired mitochondrial fatty acid oxidation capacity, but increased peroxisomal β-oxidation, the hepatic EPA may increase whereas DHA may decrease. Indeed, the hepatic level of DHA decreased after TTP treatment whereas the hepatic content of DPA, an intermediate fatty acid between EPA and DHA, increased. The hepatic level of EPA remained constant whereas the plasma concentration of both EPA and DPA increased in mice treated with TTP (Table 4). It has been reported that dietary EPA supplementation accentuates hepatic TAG accumulation in mice with impaired fatty acid oxidation capacity [26]. It was also of interest that the relative amount of long-chain SFAs, MUFAs and n-6 fatty acids decreased after TTP treatment, while the content of C12:0 and C14:0 was increased. Accordingly, impaired mitochondrial fatty acid oxidation may lead to an accumulation of medium-chain SFAs and MUFAs ≤ C18 in liver. Whether this is due to increased peroxisomal fatty acid chain shortening or increased oxidative stress [17], should be considered. Most probably this is due to increased fatty acid shortening by peroxisomes as the plasma antioxidant capacity and the hepatic gene expression of SOD1 increased (Table 3). This was unexpected as SOD1 is involved in the prevention of TAG accumulation during high-fat diet induced steatosis in mice [27]. Due to the presence of a sulfur atom in the carbon backbone, TTP potentially has antioxidant capacity, as shown for carbon-3 thia-fatty acids [28]. In further studies it will be interesting to investigate mitochondrial markers of oxidative stress, as well as the effect of TTP on oxidative phosphorylation. The almost 50 % reduction of AA resulting in an increased anti-inflammatory fatty acid index (Fig. 6) further suggested that no increased inflammation followed 2 weeks TTP administration. Accordingly the hepatic gene expression of IL-1β and TNFα decreased (Table 3). A prolonged treatment period might have resulted in monocyte recruitment and increased inflammation associated with steatohepatitis [29].

### Fatty liver and plasma total homocysteine

One-carbon metabolism is closely linked to lipid homeostasis, primarily through the role of methyl-groups in the formation of phosphatidylcholine by the phosphatidyl-ethanolamine methyltransferase (PEMT) pathway [30–33]. Methyl donor supplementation has been shown to alleviate NAFLD and reduce plasma acylcarnitines in mice [34]. The plasma Hcy level is linked to regulation of PPARs, suggesting that hyperhomocysteinemia is associated with alteration of intracellular lipid metabolism [35]. We observed a substantial increase in plasma tHcy in TTP treated animals developing fatty liver, which is in line with elevated plasma tHcy levels previously observed in NAFLD patients [36]. In the present study of TTP-induced fatty liver a strong correlation was observed between hepatic TAG concentration and plasma tHcy (Fig. 7l). These data support the view that increased levels of tHcy may be associated with hepatic steatosis at least in this model. The tHcy increase may be a consequence of reduced activity in all three pathways of Hcy metabolism as the gene expression of the rate-limiting enzyme in each pathway was significantly reduced. Decreased plasma methionine and methionine sulfoxide supports that TTP induced fatty liver is associated with a reduced hepatic methylation potential, and elevated plasma betaine specifically indicate that BHMT remethylation was low. There is currently no available biomarker for the evaluation of the remethylation associated with the folate cycle. Moreover, there were no associations between plasma levels of coenzymes, markers of B-vitamins and one-carbon metabolites (data not shown). TTP has a small PPARα activation effect [37] and PPARα activation by WY-14,643 has previously shown to increase the production of nicotinamide [38]. Thus, higher plasma nicotinamide observed in TTP-treated animals may be due to PPARα-induced synthesis. Notably, fibrate treatment was previously shown to increase plasma tHcy [39, 40], supporting a possible role of PPARα in the hyperhomocysteinemia in response to TTP treatment.

### Fatty liver and metabolites of the choline oxidation pathway

In line with a possible reduced methylation potential in TTP-treated animals, plasma L-carnitine, requiring 3 methylation steps to be produced, was significantly reduced. However, the plasma concentration of 6-N-trimethyllysine, a methylated amino acid and L-carnitine precursor, did not decrease in TTP-induced fatty liver. Glycine, generated during L-carnitine biosynthesis, was not altered in plasma. Glycine can also be produced from serine in the cell cytosol and mitochondria via SHMT1 and SHMT2, respectively (Fig. 1), and the gene expression of mitochondrial SHMT2 was downregulated. Sarcosine, which was significantly increased by TTP, can be produced from glycine in the cell cytosol via GNMT. The gene expression of GNMT was, however, unchanged. Finally, glycine can be produced from sarcosine by the mitochondrial B2-dependent SARDH enzyme, which was downregulated, suggesting that reduced sarcosine catabolism may have contributed to the higher plasma concentration of sarcosine during TTP-induced fatty liver. Moreover, the concentration of the mitochondrial metabolite DMG was increased after TTP administration, despite a reduction in the gene expression of *Bhmt*. The increased concentration of DMG in TTP-induced fatty liver could be explained by decreased catabolism of DMG, in line with the observed reduction in *Dmgdh* gene expression.

## Conclusions

Our results suggest that the hepatic steatosis induced by the modified fatty acid analogue TTP is most likely due to inhibited mitochondrial fatty acid oxidation capacity, and is associated with accumulation of long-chain L-carnitine derivatives and altered hepatic fatty acid composition. We demonstrate that TTP-induced mitochondrial dysfunction and hepatic steatosis can influence tHcy levels and one-carbon metabolites without an increase in oxidative stress. Because TTP affected B-vitamin status and gene expression of enzymes involved in choline oxidation and Hcy metabolism, the influence of one-carbon and Hcy metabolism on hepatic steatosis development should be further evaluated.

## Methods

### Animals

Male C57BL/6 mice (Taconic, Denmark), 12 weeks old, were housed in Makrolon III cages, 2-3 animals per cage, in an open system. They were kept under standard laboratory conditions with temperature 22 ± 1 °C, dark/light cycles of 12/12 h, relative humidity 55 ± 5 % and 20 air changes per hour. The animal study was conducted according to the Guidelines for the Care and Use of Experimental Animals, and the Norwegian State Board of Biological Experiments with Living Animals approved the protocol (FOTS ID: 2013/5077).

Animals were divided at random into 4 groups of 8 mice, and after 7 days of acclimatization, control groups were fed a diet with 7 % (w/w) fat (5 % lard and 2 % soy oil, Dyet Inc., Bethlehem, PA, USA) while the intervention diets were supplemented with 0.3 %, 0.6 % or 0.9 % TTP. TTP was manufactured as previously described [41]. Isoenergetic diets were made as described in Table 5, containing 19 % (w/w) protein from casein, cornstarch, dyetrose, sucrose, fiber, AIN-93-MX mineral mix, AIN-93-VX vitamin mix, L-cysteine, choline bitartrate (Dyet Inc.) and tert-Butyl-hydroquinone (Sigma Aldrich, St. Louis, MO, USA). All groups had free access to tap water and feed during the 14 days experiment. Mice feed intake and weight gain were determined twice a week. All mice were killed on day 14. They were anesthetized by inhalation of 2 % isofluorane (Forane, Abbot Laboratories Ltd, Illinois, USA) in an anesthesia chamber and thoracotomy, cardiac puncture, and exsanguination was performed. Plasma and snap-frozen liver samples were stored at -80 °C.

**Table 5:** Composition of the experimental diet^a^

Diet component	Amount (g)
Lard	5.0
Soy oil	2.0
Casein^b^	22.0
Cornstarch	38.8
Dyetrose	12.9
Sucrose	9.8
Fiber	4.9
AIN-93G-MX mineral mix	3.4
AIN-93-VX vitamin mix	4
L-Cystine	0.3
Choline bitartrate	0.2
Tert-butyl-hydroquinone	0.0014

^a^Values shown are g component per 100 g diet. The energy contribution from protein, fat, and carbohydrates was 19.5, 16, and 64.5 %, respectively, and 100 g diet contained 381.4 kcal. TTP was added to the complete diet at a dose of 0.3, 0.6 or 0.9 g per 100 g

^*b*^The casein contained 86.5 % protein

### Hepatic enzyme activities

At sacrifice, livers were removed, and weighed. One part of the liver was immediately snap-frozen in liquid nitrogen, while 100 mg liver from each mouse was chilled on ice and homogenized in 1 mL ice-cold sucrose medium (0.25 M sucrose, 10 mM HEPES, and 1 mM Na_4_EDTA, adjusted to a pH of 7.4 with KOH) giving 10 % (w/v). The homogenates were centrifuged at 600 g-force for 10 min at 4 °C and the post-nuclear fraction was removed and used for further analysis. The mitochondrial fatty acid oxidation in the absence and presence of malonyl-CoA was assayed as previously described [42].

The assay for CPT I and II was performed on frozen homogenate according to Bremer et al. [43], but with some modifications [44]. The activity of ACOX1 was measured in the post-nuclear fraction, as described by Madsen et al. [25]. FAS and GPAT activities were measured in the post-nuclear fraction as described by Skorve et al. [45] with some modifications [44]. ACSL activity was measured according to Bar-Tana, Rose and Shapiro [46]. HMG-CoA synthase was measured as described by Clinkenbeard et al. [47]. Citrate synthase was determined according to Stadlmann et al. [48].

### Histology

Cryo-sections from frozen livers were generated using a 1720 Cryostat (Leica Microsystems, Wetzlar, Germany) from 3 mice per group. Sections were fixed in 4 % buffered formalin for 10 min, rinsed 3× in dH_2_O, before staining in 0.7 % (w/v) Oil Red O (Sigma) in propylene glycol for 10 min, rinsed 3× dH_2_O, and stained with hematoxylin (Thermo Fisher Scientific, Waltham, MA, USA) for 2 min. Finally, sections were rinsed 3× dH_2_O and mounted with ImmuMount (Thermo Fisher Scientific). Images were captured using an Olympus BX51 light microscope at 40× magnification with an Olympus DP25 digital colour camera (Olympus Corporation, Tokyo, Japan). Three images were captured from each animal by a blinded investigator.

### Biochemical analyses

Liver lipids were extracted according to Bligh and Dyer [49], evaporated under nitrogen, and redissolved in isopropanol before analysis. Lipids from liver extracts or plasma were then measured enzymatically on a Hitachi 917 system (Roche Diagnostics GmbH, Mannheim, Germany) using the TAG (GPO-PAP) and cholesterol kit (CHOD-PAP) from Roche Diagnostics, and the free fatty acid kit and phospholipid kit from Diasys Diagnostic systems GmbH (Holzheim, Germany). A kit from Roche was used to determine the plasma activities of ALT. Total liver fatty acid composition was analyzed in liver extracts and plasma using GC/MS as previously described [50]. The DBI was calculated according to the formula: (total MUFA + 2*(C18:2n-6 + C20:2n-6 + C22:2n-6) + 3*(C18:3n-6 + C18:3n-3 + C20:3n-9 + C20:3n-6) + 4*(C18:4n-3 + C20:4n-6 + C22:4n-6) + 5*(C20:5n-3 + C21:5n-3 + C22:5n-6 + C22:5n-3) + 6*C22:6n-3)/total fatty acids. The anti-inflammatory fatty acid index was calculated according to the formula: ((20:5n-3 + 20.3n-6 + 22:6n-3/20:4n-6)) × 100.

Free carnitine, acetyl-, octanoyl-, palmitoyl-, propionyl-, and (iso)valerylcarnitines, and the precursors for carnitine, butyrobetaine and trimethyl lysine, were analyzed in plasma using LC MS/MS as previously described [51] with some modifications [52]. Plasma methylmalonic acid, tHcy, sarcosine, serine and glycine were analyzed by GC-MS/MS [53]. Plasma choline, betaine, DMG, methionine, methionine sulfoxide, cysteine, [54], all vitamin B2, B3, and B6 forms [flavine mononucleotide, riboflavin, nicotinamide, N1-methylnicotinamide, pyridoxal 5′ phosphate, pyridoxal, 4-pyridoxic acid], and cystathionine [55] were analyzed by LC-MS/MS at Bevital A/S (http://www.bevital.no; Bergen, Norway) according to Midttun, Hustad and Ueland [55]. Total antioxidant capacity of plasma was measured using the total antioxidant capacity kit (Abcam, Cambridge, UK) according to the manufacturer’s instructions.

### Gene expression analysis

Total cellular RNA was purified from frozen liver samples, and cDNA was produced as previously described [56]. Real-time PCR was performed with Sarstedt 384 well multiply-PCR Plates (Sarstedt Inc., Newton, NC, USA) on the following genes, using probes and primers from Applied Biosystems: *Chka* (Mm00442759_m1), *Chkb* (Mm04213225_s1), *Chdh* (Mm00549261_m1), *Bhmt* (Mm04210521_g1), *Dmgdh* (Mm00512022_m1), *Sardh* (Mm00454657_m1), *Shmt1* (Mm00486110_m1), *Shmt2* (Mm00659512_g1), *Gnmt* (Mm00494688_m1), *Mtr* (Mm01340053_m1), *Mtrr*_(Mm00549978_m1), *Msra* (Mm00452738_m1), *Msrb2* (Mm00512937_m1), *Cbs* (Mm00460654_m1), *Cth* (Mm00461247_m1), *Ppargc1a* (Mm00447183), *Pparg* (Mm00440945), *Ppara* (Mm00440939), *CD36*/*Fat* (Mm00432403), *Fabp1* (Mm00444340), *Lipe* (Mm00495359), *Acly* (Mm01302282_m1), *Slc25a20* (Mm00451571_m1), *Lipc* (Mm00433975), *Apob* (Mm01545156_m1), *IL1β* (Mm01336189_m1), *Sod1* (Mm01344233_m1), *Sod2* (Mm01313000_m1), *Tnfα* (Mm00443258_m1) and *Pcyt1a* (Mm00447774_m1).

Three different reference genes were included: *18 s* [Kit-FAM-TAMRA (Reference RT-CKFT-18 s)] from Eurogentec, Belgium, glyceraldehyde-3-phosphate dehydrogenase (*Gapdh*, Mm99999915_g1) from Applied Biosystems, and ribosomal protein, large, P0 (*Rplp0*, Gene ID 11837) from Thermo Fisher Scientific. The NormFinder software was used to evaluate the reference genes [57], and data normalized to *18 s* are presented.

### Statistical analysis

Data was analyzed using Prism Software (Graph-Pad Software, San Diego, CA) to determine statistical significance. The results are shown as means of 7-8 animals per group with their standard deviations. Plasma samples were pooled to obtain enough material, and three samples per group were analyzed, as indicated in figure legends. Normal distribution was determined by the Kolmogorov-Smirnov test (with Dallal-Wilkinson-Lilliefor *P* value), when possible. One-way ANOVA with Dunnett’s post hoc test was performed to evaluate statistical differences between groups. Unpaired *t*-test was used to analyze gene expression results, and to determine tendencies. *P*-values < 0.05 were considered statistically significant.
